# Supplementary material for: Gene co-expression network analysis to identify critical modules and candidate genes of drought-resistance in wheat
Source: PLoS One. 2020 Aug 31;15(8):e0236186. doi: 10.1371/journal.pone.0236186 (PMC7458298; doi:10.1371/journal.pone.0236186)
Supplement: S1 Table — (DOCX) [file pone.0236186.s001.docx]

S1 Primers of genes used in RT-qPCR

| Gene ID | Forward primer sequence | Reverse primer sequence |
| --- | --- | --- |
| TraesCS7D01G417600 | GATGTGGGCAGGTCAGTGTCT | GTCACCAAATGCCCGAGAAA |
| TraesCS5B01G565300 | GAGCTTGAGGACCTTGGTACAGA | CGATCAGTCCATCATCTTCAAGTC |
| TraesCS4A01G068200 | CGGTAAGTCCTCGTTCCTGATG | GGGTCCACTAGCCCTGCATT |
| TraesCS2D01G033200 | TTGCTGAATCCCTGAATGAATG | GCCACGGCAAACAATCATC |
| TraesCS6B01G425300 | TGCCTGACACTATCACTGAGTACATG | GGTTGCTACATTCTGCATTCCA |
| TraesCS7A01G499200 | CTCCGCAACAAGCCAGTGA | AGCCCCAAGGTACTCCATCA |
| TraesCS4A01G118400 | TTTCTCTGACACACCATCGTTATCA | CAGTGACCCGTGAGCCAGAT |
| TraesCS2B01G415500 | GGCCGATGAGGCACCTTATT | AATCAGACAGGTGTGGCTCAAA |
| TraesCS1A01G129300 | GCATAGGTGGCAGAAGGTTTTG | ACCATTTCGTGGGTCCATATTT |
| TraesCS2D01G326900 | AATGGCAGCATCTCGGAAAA | GACACAGATGACTGGTCCAAAGAC |
| TraesCS3D01G227400 | CTGCTGATTGTGATTTTGTTTGG | GGGCTTTTGACCGTACTTTCG |
| TraesCS3B01G144800 | GAATGCCACCAATCGAGAGAA | GCCGAACCAGTATCAAGGAACA |
