# Supplementary material for: Gene co-expression network analysis to identify critical modules and candidate genes of drought-resistance in wheat
Source: PLoS One. 2020 Aug 31;15(8):e0236186. doi: 10.1371/journal.pone.0236186 (PMC7458298; doi:10.1371/journal.pone.0236186)
Supplement: Attachment — Submitted filename: Response to Reviewers.docx [file pone.0236186.s002.docx]

Dear Dr. Ma

Thank you very much for handling our manuscript and allowing us to revise our manuscript (Manuscript Number: PONE-D-20-08497). We also thank the reviewers who provided many constructive suggestions on our manuscript. We have studied editor and reviewer’s comments carefully and have made revision which marked in red in the manuscript and describe all the revised details in point-by-point response as follows:

**Revision – authors’ response**

**Editor:**

We want to thank the editor for constructive and insightful criticism and advice. We addressed all the points raised by the editor as summarized below.

1. We have modified the manuscript to meet PLOS ONE's style requirements

2. In the methods section, we have provided additional details regarding the plant materials and described the source.

3. We have completely revised the language use, spelling, and grammar of the manuscript.

4. We have uploaded these data to NCBI Sequence Read Archive, and the relevant accession numbers used to access these data have shown in lines 173-174.

5. We have registered an ORCID iD as required.

6. We have added a description of Table 1in line 232.

**Referee #1:**

We want to begin by thanking Referee #1 for writing that “Drought stress is an important limiting factor in wheat production. The study is interesting.” We also appreciated the constructive criticism and suggestion. We addressed all the points raised by the reviewer, as summarized below.

1. Describe the treated samples at which growth stages, which tissues in Material, and Figure1 should explain the meanings of C vs A, CR VS DR, ... (in results).

As the reviewer suggested, in the material method section, we added a description of the growth stage and tissue of the treated samples and added the meaning interpretation of C vs A, CR vs DR… in the legend of Figure 1 on the result section.

2. Thanks to the referee’s comment, we have changed the “drought-resistant“ to “drought-resistance“ in the title.

3. In Abstract and result parts, in the "genes involved in the modules, such as dark turquoise, yellow and brown, were found", dark turquoise, yellow and brown should be explained by expert names, such as detailed genes/pathways.

Thank you very much for your comments. In the abstract and results section, the module's names were described in dark turquoise, yellow and brown ... This is because when WGCNA is used for gene clustering analysis, genes that are functionally related or similar are grouped and represented by the same color. The same color module contains many genes, which cannot be enriched into a single pathway or explained by expert names. So only colors can be used to describe modules.

4. In Abstract, Twelve central, greatly correlated genes in stage-specific modules were subsequently confirmed and validated at the transcription levels, including TraesCS7D01G417600.1, TraesCS5B01G565300.1, TraesCS4A01G068200.1, TraesCS2D01G033200.1, TraesCS6B01G425300.1, TraesCS7A01G499200.1, TraesCS4A01G118400.1, TraesCS2B01G415500.1, TraesCS1A01G129300.1, TraesCS2D01G326900.1, TraesCS3D01G227400.1 and TraesCS3B01G144800.1, the genes should be written with the gene name, such as PP2C, ERF1B...

As the reviewer suggested, We looked up the names of these genes and added them in parentheses.

5. Lines 26-27, and results, what are five of the genes newly identified for drought resistance in the study? How do you verify their functions in drought resistance?

Referee #2 asks what are five of the genes and how to verify….. Lines 351-353, we added the specific names of these five genes to the paper. We treated wheat Jimai 418 in the seedling stage with drought (20%PEG) for a different time and extracted RNA for RT-PCR verification. It was found that the expression levels of these five genes were significantly different under drought stress, which was speculated to be related to response to drought stress. We would like to note that we investigated the five genes in the previous study and found no reported that the five specific genes were associated with drought resistance in wheat. Therefore, in this manuscript, we focused on these five genes and will carry out transgenic verification in subsequent experiments.

6. The MS English writing must be carefully improved and thoroughly edited by a native English speaking expert.

As suggested by the reviewer we have invited professional scientific editing service (peerwith) and native expert (Ally Oakes) to polish the language of the article. Please see the attachment for the editing certificate. Our colleagues studying abroad have also made careful improvements to the English expressions throughout the manuscript. All changes have been revised in the manuscript with track changes.

7. Because of the referee’s comment, we have numbered the pictures in the manuscript.

**Referee #2:**

We would like to thank the referees for their thoughtful review of our manuscript. We believe that the additional changes we have made in response to the reviewer's comments have made this a significantly stronger manuscript. Below is our point-by-point response to the referee’s comments.

Major issues

1. The English language needs professional proofreading as there are massive typos and grammar errors in the main document.

The language of the article has been polished by professional scientific editing service (peerwith) and native expert (Ally Oakes). Please see the attachment for the editing certificate. Our colleagues studying abroad have also made careful improvements to the English expressions throughout the manuscript. All changes have been revised in the manuscript with track changes.

2. A short introduction referring to the current understanding of drought-resistant in wheat is suggested.

According to the referee’s suggestion, we added a brief introduction to the current understanding of drought-resistant in wheat at the end of the first paragraph.

3. A total of twelve genes (TraesCS7D01G417600.1, TraesCS5B01G565300.1, TraesCS4A01G068200.1, TraesCS2D01G033200.1, TraesCS6B01G425300.1, TraesCS7A01G499200.1, TraesCS4A01G118400.1, TraesCS2B01G415500.1, TraesCS1A01G129300.1, TraesCS2D01G326900.1, TraesCS3D01G227400.1 and TraesCS3B01G144800.1) were validated at the transcription levels, why?

According to the k-mean diagram of 9 modules determined in this paper, the drought-treated tissues and developmental stages were compared with the transcriptional results, and eight modules with consistent results were obtained for analysis. The 38 genes with the highest correlation degree were selected by using the protein interaction diagram. After consulting the gene function and referring to previous literature, we speculated that these 12 genes might be related to drought.

Minor issues

1. Thanks to the referee’s comment, the wrong background have removed in the text.

2. Line105, the “follow-up test material” was changed to “test material”.

3. Line 110, to better understand we have punctuated in this sentence.

4. lines 108-109, we agree with the reviewer's comment and changed to “The samples were collected from 3 separate plants and pooled for RNA extraction.”

5. Line 150, according to the referee’s suggestion, 2-ΔΔCT was changed to 2^-ΔΔCT^.
